# Controllable Synthesis of Manganese Organic Phosphate with Different Morphologies and Their Derivatives for Supercapacitors

**DOI:** 10.3390/molecules29174186

**Published:** 2024-09-04

**Authors:** Jingwen Zhao, Qingling Jing, Ting Zhou, Xinhuan Zhang, Wenting Li, Huan Pang

**Affiliations:** School of Chemistry and Chemical Engineering, Yangzhou University, Yangzhou 225009, China; 13773809781@163.com (J.Z.); jql971122lh@163.com (Q.J.); 18251947900@163.com (T.Z.); 19352670640@163.com (X.Z.)

**Keywords:** metal-organic framework, manganese organic phosphate, manganese compounds, supercapacitor, solid-state device

## Abstract

Morphological control of metal-organic frameworks (MOFs) at the micro/nanoscopic scale is critical for optimizing the electrochemical properties of them and their derivatives. In this study, manganese organic phosphate (Mn-MOP) with three distinct two-dimensional (2D) morphologies was synthesized by varying the molar ratio of Mn^2+^ to phenyl phosphonic acid, and one of the morphologies is a unique palm leaf shape. In addition, a series of 2D Mn-MOP derivatives were obtained by calcination in air at different temperatures. Electrochemical studies showed that 2D Mn-MOP derivative calcined at 550 °C and exhibited a superior specific capacitance of 230.9 F g^−1^ at 0.5 A g^−1^ in 3 M KOH electrolyte. The aqueous asymmetric supercapacitor and the constructed flexible solid-state device demonstrated excellent rate performance. This performance reveals the promising application of 2D Mn-MOP materials for energy storage.

## 1. Introduction

Metal-organic frameworks (MOFs) possess distinct advantages, including the uniform distribution of metal centers and adjustable functional groups for various applications [1,2,3,4,5,6]. Two-dimensional (2D) MOFs with exceptional specific surface area (SSA) and ordered porosity are more favorable for ion migration than common porous carbon or zeolite materials [7,8,9]. Given the focus on energy issues, 2D MOFs are recognized as promising materials for supercapacitor (SC) electrodes due to their unique structural characteristics [10,11]. The performance of SCs is significantly influenced by the choice of electrode materials depending on their working principle. However, the low electrical conductivity of most MOFs limits their application in SCs [12,13,14,15]. An effective strategy is to adjust the geometric size and morphology of MOFs, which address these issues and provide opportunities to integrate new properties and functions while preserving their original characteristics.

Metal organophosphates (MOPs) provide both carbon and phosphorus sources at a molecular level, facilitating the introduction of conductive components into metal phosphides. Compared to oxides, hydroxides, sulfides, and phosphides, metal phosphates with non-metal PO_4_^3−^ anions typically offer better electrochemical performance due to their high electrochemical activities, non-toxicity, and chemical stability. The strong P-O covalent bonds boost the redox potential and improve the chemical structure stability of manganese phosphate [16,17]. For enhancing the performance of SCs, it is essential to have a chemically stable structure and short diffusion path length of the charge carrier electrode. MOPs have become a momentous part of MOFs-based materials [18]. However, many ligands in the MOP framework are insulators, bringing about inferior conductivity [19,20]. Moreover, these large-sized bulk materials have poor stability and are prone to decomposing in strong acid/alkali systems. Consequently, modifying MOP materials for practical applications has attracted wide attention. Heat treatment is a simple and scalable method to obtain MOP derivatives (carbon-based materials or metal compounds) [21,22]. MOP derivatives typically exhibit excellent chemical and physical properties due to the presence of the retaining structure of the pristine MOP [23]. The size and morphology of MOP derivatives greatly influence their properties, making morphology control a research hotspot [24,25,26]. The micro/nano structure of MOFs can be effectively adjusted by altering reaction conditions, such as reaction temperature, reaction time, pH value, reactant molar ratio, and the type and amounts of surfactants and regulators.

Herein, a simple and effective calcination strategy was proposed to prepare a series of manganese organic phosphate (Mn-MOPs) samples as electrode materials for SCs. The Mn-MOP derivatives with different morphologies were obtained by adjusting the molar ratio of metal ions to ligands. As the molar ratio changed from 1:3, 3:5 to 1:1, the morphology of Mn-MOF changed from a palm leaf and nanometer strip to a nanometer sheet. The Mn-0.05-550 product showed better electrochemical performance with a specific capacitance of 230.9 F g^−1^ at 0.5 A g^−1^ in 3 M KOH. Moreover, the performance of Mn-MOP derivatives in flexible solid-state SC was also excellent. This work proposed a novel concept of MOP materials for energy storage.

## 2. Results and Discussion

### 2.1. Morphological and Structural Analysis

**Figure 1:**
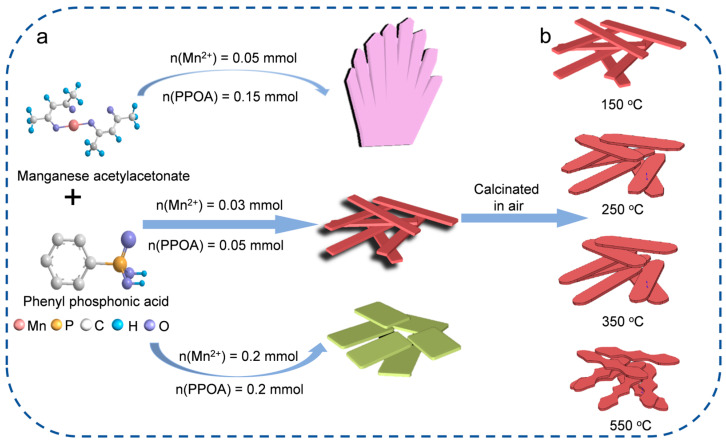
(**a**) Schematic synthesis of Mn-MOP; (**b**) Mn-MOP precursors’ calcination route.

The synthesis of Mn-MOP of different morphologies and their derivates is shown in Figure 1a. Two-dimensional nanomaterials with three different morphologies were prepared by a simple one-step solvothermal reaction of manganese(Ⅱ) acetylacetonate (C_10_H_14_MnO_4_) and phenyl phosphonic acid in N,N-dimethylformamide (DMF) solution, with C_10_H_14_MnO_4_ as a source of metal ions and phenyl phosphonic acid as a connector support. The influence of the molar ratio of reactants on their morphologies was also studied. Mn-MOPs with three different morphologies were obtained only by changing the molar ratio of Mn^2+^ to phenyl phosphonic acid. A molar ratio of 1:3 results in the palm leaf morphology, which is designated as Mn-DMF-0.15. When the molar ratio is 3:5, the morphology of the product becomes a nanoscale strip, which is named Mn-DMF-0.05. When the molar ratio is 1:1, the morphology of the product becomes a nanosheet, called Mn-DMF-0.2. Figure 1b illustrates the calcination path of Mn-DMF-0.05 derivatives. The products obtained after calcination are named Mn-0.05-x, where x refers to the calcination temperature.

**Figure 2:**
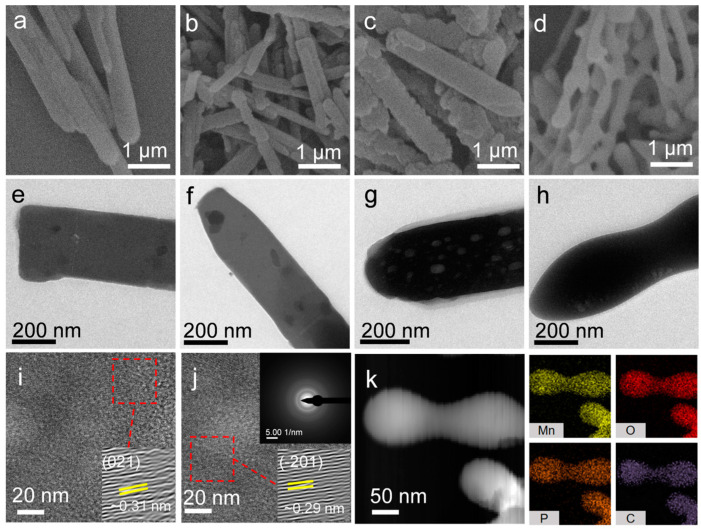
(**a**–**h**) SEM and TEM images of Mn-0.05-x (x = 150, 250, 350 and 550 °C); (**i**,**j**) HRTEM image (inset: SAED); (**k**) EDX mapping results of Mn-0.05-550.

According to the electrochemical performance, Mn-DMF-0.05 was used as the precursor for calcination. The thermogravimetric analysis results showed that double main weight loss occurred at 20 to 900 °C (Figure S3). The first apparent weight loss stage (150–180 °C) resulted from the disappearance of crystalline water. Due to the gradual decomposition of the organic group, the second weight loss stage occurred between 232 and 580 °C. When the calcination temperature was more than 580 °C, the organic group decomposed completely. Therefore, we chose the four temperatures (150, 250, 350, and 550 °C) for calcination in the air atmosphere. The SEM and TEM images display the overall morphology changes of the Mn-DMF-0.05 after treatment at different calcination temperatures (Figure 2 and Figure S1). The Mn-0.05-150, Mn-0.05-250, and Mn-0.05-350 samples with an average length of 3–5 μm maintain their nano-strip morphologies (Figure 2a–c), and some pores could be observed in Mn-0.05-350 (Figure 2e–g). The Mn-0.05-550 sample maintains its partial nano-strip morphology as shown in Figure 2d,h. The morphology of the Mn-0.05-550 maintains the nano-strip shape, and the edges become round. The interplanar spacing of 0.31 and 0.29 nm is consistent with that of (021) and (−201), respectively. The selected area electron diffraction (SAED) patterns prove a good crystallinity of Mn-0.05-550 (Figure 2i,j). The energy dispersive X-ray (EDX) results of Mn-0.05-550 demonstrated that Mn, O, C, and P were distributed throughout the nanoribbon (Figure 2k). It can be concluded that the morphology and spatial structure of Mn-MOP derivatives changed obviously after treatment at a calcination temperature of 550 °C.

**Figure 3:**
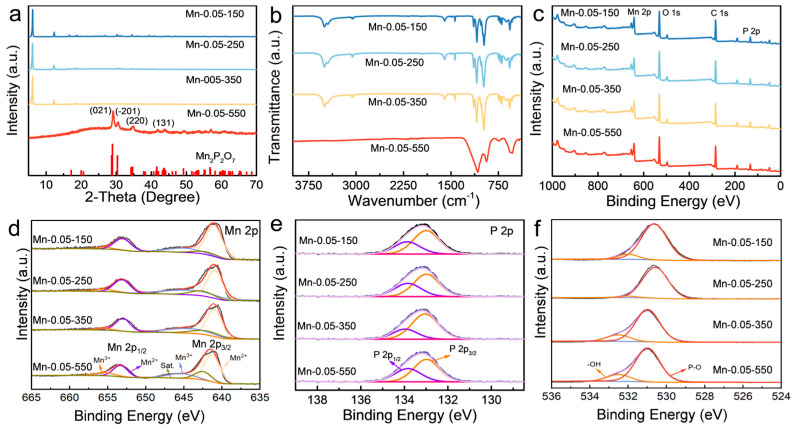
(**a**) XRD patterns of Mn-0.05-x; (**b**) FT-IR spectra of Mn-0.05-x; (**c**) full XPS spectrum of Mn-0.05-x; (**d**–**f**) Mn 2p, P 2p, and O 1s XPS spectra of Mn-0.05-x.

The XRD patterns of the Mn-MOP derivatives are displayed in Figure 3a and Figure S2. The crystal structure of Mn-MOP derivatives was consistent with that of Mn-MOP, when the calcination temperature was lower than 350 °C. Mn-MOP became manganese pyrophosphate (Mn_2_P_2_O_7_) when the calcination temperature was 550 °C. The main peaks of Mn-0.05-550 appeared at 28.9°, 30.3°, 34.6°, and 41.5°, which correspond to the (021), (−201), (220), and (131), respectively (PDF#29-0891-550). According to the FT-IR spectra, the functional groups of the Mn-MOP precursor are preserved in samples calcined at relatively low temperatures. Meanwhile, Mn-0.05-550 displays strong peaks at 937.2 and 1076.1 cm^−1^, which results from the vibration absorption of the P-O bond. The apparent signal at ~555.4 cm^−1^ arises from Mn-O. The stretching vibration peak of the benzene ring disappeared because the Mn-MOP precursor is calcined at 550 °C, which demonstrates that Mn-MOP was entirely decomposed, resulting in the formation of Mn_2_P_2_O_7_. These results obtained by FT-IR are consistent with those of the XRD results (Figure 3b). The chemical states of the individual Mn, O, C, and P elements in these samples are determined by XPS. The main elemental components of Mn-MOP derivatives are Mn, O, C, and P (Figure 3c). A broad peak for Mn 2p of Mn-0.05-550 depicted in Figure 3d is divided into several binding energy regions. The two main peaks at 641.3 eV and 653.4 eV correspond to Mn 2p_3/2_ and Mn 2p_1/2_, respectively. This is a typical XPS signature of Mn^2+^, confirming the presence of Mn^2+^ in the sample [27]. In addition, a few portion peaks at 645.1 eV, 655.4 eV, and 642.5 eV can be observed, corresponding to Mn^3+^ (Mn 2p_3/2_ and Mn 2p_1/2_, respectively) and its satellite peak [28,29]. In addition, the P 2p spectrum exhibits two representative peaks at 132.99 eV and 133.84 eV (Figure 3e), indicating different chemical environments, i.e., PO_4_^3−^ and PO_3_^−^ [30]. The XPS spectrum of O 1s displays peaks at 531.0 eV and 532.6 eV, corresponding to the P-O and -OH bonds, respectively (Figure 3f) [27]. In general, manganese pyrophosphate is the primary component of the MOP derivatives, with Mn^2+^ being the predominant oxidation state in the sample. It is observed that the as-prepared Mn-0.05-550 had a specific surface area of 130.4 m^2^ g^−1^, as displayed in Figure S4a. The pore-size distribution of Mn-0.05-550 was examined using the Barret–Joyner–Halenda (BJH) method. Remarkably, the prepared Mn-0.05-550 possesses maximum pores of 2–10 nm (Figure S4b), suggesting a mesoporous nature, which can assist efficient ion diffusion and charge transport.

### 2.2. Electrochemical Performance Studies

**Figure 4:**
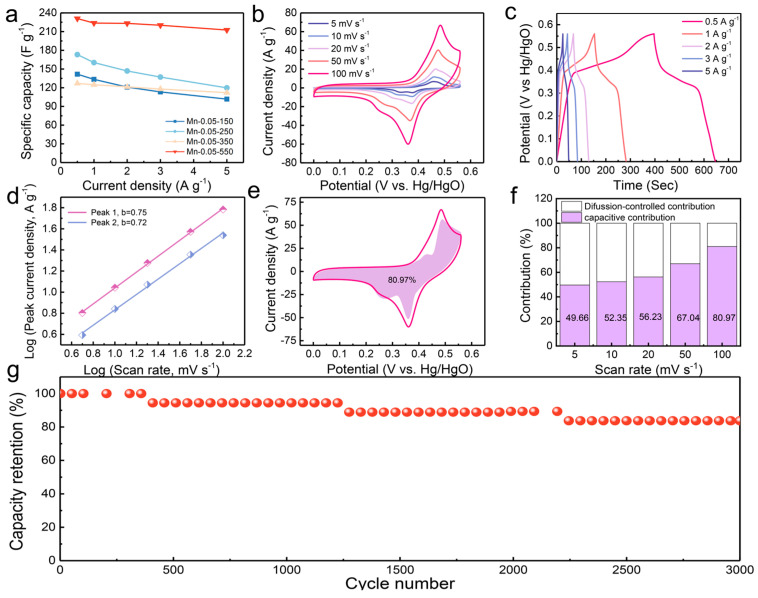
(**a**) Rate capability of Mn-0.05-x; (**b**) CV profiles of Mn-0.05-550 at various scan rates; (**c**) GCD profiles of Mn-0.05-550; (**d**) log(*i*) versus log(*v*) plots of Mn-0.05-550; (**e**) capacitive contribution of Mn-0.05-550 at 100 mV s^−1^; (**f**) percentages of capacitive contributions of Mn-0.05-550; (**g**) cyclic performance of Mn-0.05-550 at 4 A g^−1^.

The electrochemical performances of Mn-MOP and Mn-0.05-x were evaluated in a three-electrode system. The Mn-0.05-x electrodes exhibited clear redox peaks in the cyclic voltammetry (CV) curves at varied scan rates and potentials (Figure 4b and Figures S5 and S6). These results demonstrate that the Mn-MOP and Mn-0.05-x electrodes show faradaic pseudocapacitive behavior. Among them, the peak current of the Mn-0.05-550 electrode is higher than that of other samples, showing a significantly enhanced electrochemical activity [31].

In addition, this work investigates the charge transfer kinetics of Mn-0.05-550 by CV at different scan rates (Figure 4d). These results show that there is both a diffusion control process and a surface capacitance control process upon cycling [32]. The capacitance contributions are calculated as shown in Figure 4e,f and Figures S7–S12. The capacitance contributions increase with the increase in scan rates, which indicates that the charge storage efficiency is high. Compared with other electrodes, Mn-0.05-550 has a higher capacitive contribution, which is proved by galvanostatic charge–discharge (GCD) tests. According to the GCD curves (Figure 4a,c and Figure S13), Mn-0.05-550 has the highest specific capacitance. The specific capacitances of Mn-0.05-550 are achieved at 230.9, 223.7, 223.2, 220.2, and 212.5 F g^−1^ at 0.5, 1, 2, 3, and 5 A g^−1^, respectively. The cycle stability of the Mn-0.05-550 electrode was tested at 4 A g^−1^ (Figure 4g). The capacitance retention of Mn-0.05-550 is approximately 84% at the 3000th cycle. The decrease of the specific capacitance results from the collapse of Mn-0.05-550 nanosheets upon cycling [33,34]. As shown in Figure S14, the Mn-0.05-550 sample exhibits a slope greater than that of all other electrode samples. The result indicates that a lower ion diffusion resistance can achieve superior supercapacitive performance [35]. In addition, a comparative analysis of the electrochemical performance of the Mn-0.05-550 electrode against other manganese-based compounds is presented (Table S1). According to the comparison results, it was demonstrated that the Mn-0.05-550 electrode demonstrates either superior or comparable specific capacitance compared to previous studies, exhibiting its excellent electrochemical performance.

**Figure 5:**
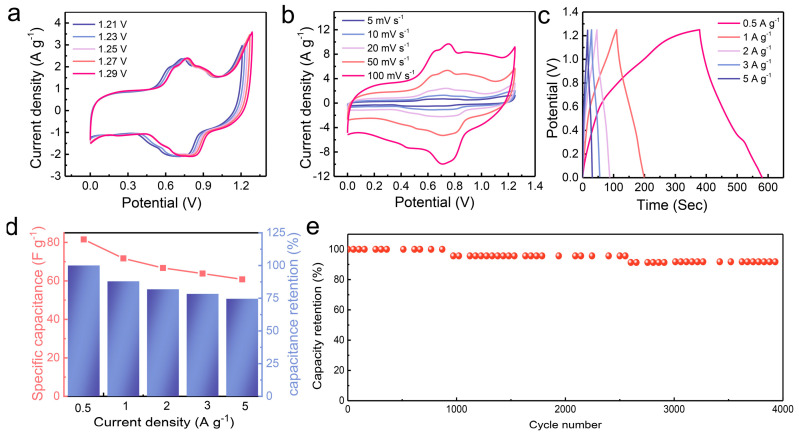
Electrochemical performance of the Mn-0.05-550//AC aqueous device. (**a**) CV profiles at varied voltages; (**b**) CV curves at varied scan rates; (**c**) GCD profiles at different current densities; (**d**) bar chart of specific capacitance and capacitance retention; (**e**) cyclic performance at 4 A g^−1^.

The aqueous asymmetric SC is assembled by using Mn-0.05-550 and AC as the positive and negative electrodes. The CV curves for the Mn-0.05-550//AC device at various scan rates and voltages are presented in Figure 5a,b. Moreover, even at 100 mV s^−1^, the CV profile of the Mn-0.05-550//AC device is consistently maintained, demonstrating that the Mn-0.05-550//AC aqueous-based device exhibits superb rate capability. The GCD curves of Mn-0.05-550//AC (Figure 5c) were investigated, and the specific capacitances are depicted in Figure 5d. When the current density increased, the capacitances of Mn-0.05-550//AC devices were 81.6, 71.7, 66.7, 63.8, and 60.8 F g^−1^, respectively, with capacitance retention values of 100, 87.9, 81.8, 78.2, and 74.6%, respectively. As shown in Figure 5e, we tested the cycle stability of the Mn-0.05-550//AC device. At the 4000th cycle, the capacitance retention of the Mn-0.05-550//AC aqueous device was 92% of the initial capacity at 4 A g^−1^.

**Figure 6:**
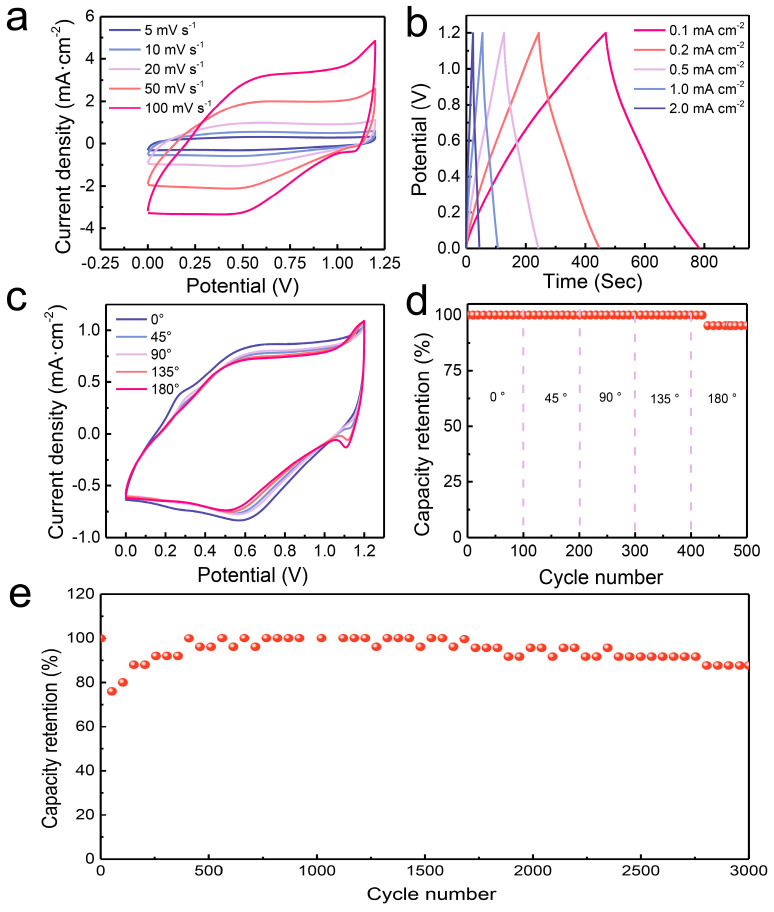
Electrochemical performance of the Mn-0.05-550//AC flexible solid-state device. (**a**) CV profiles at various scan rates; (**b**) GCD profiles; (**c**) CV profiles under various bending conditions; (**d**) cyclic stability under different bending conditions; (**e**) cyclic performance at 3 mF cm^−2^.

We also assembled the flexible solid-state device. The shapes of each CV curve maintained the original shapes with the increase in scan rates ranging from 5 mV s^−1^ to 100 mV s^−1^, demonstrating the superb rate capability of the Mn-0.05-550//AC solid-state device (Figure 6a). Figure 6b displays the GCD profiles of the Mn-0.05-550//AC device at varied current densities, and the corresponding specific capacitances were 52.50, 51.00, 48.33, 43.00, and 35.67 mF cm^−2^ at 0.2, 0.3, 0.5, 1, and 2 mA cm^−2^ (Figure S15). The flexibility of the Mn-0.05-550//AC-based solid-state device was assessed by testing it under different bending conditions. The Mn-0.05-550//AC-based device showed a loss of 4.8% after 500 cycles when bending (Figure 6c,d). We also tested the stability of the Mn-0.05-550//AC flexible solid-state device at 3 mA cm^−2^. The capacitance retention of ~87.7% was achieved at the 3000th cycle (Figure 6e). An increase of the capacity retention could be observed up to 500 cycles, which can be ascribed to the activation process of the Mn-0.05-550//AC device [36]. These results above show that the Mn-0.05-550//AC device shows promise for practical applications.

## 3. Materials and Methods

### 3.1. Materials

All the chemicals in this study, including C_10_H_14_MnO_4_, phenylphosphonic acid, DMF, Nickel foam, were used as received without further purification. All aqueous solutions were freshly prepared with high-purity water (18 MΩ cm^−1^).

### 3.2. Materials Synthesis

#### 3.2.1. Synthesis of Mn-DMF-0.15

C_10_H_14_MnO_4_ (0.05 mmol) and C_6_H_7_O_3_P (0.15 mmol) were dissolved in 5 mL DMF and stirred at room temperature. The mixture was dispensed into a Teflon-lined stainless-steel autoclave. The autoclave was maintained at 120 °C for 12 h, and then naturally cooled to room temperature. The resulting precipitate was thoroughly washed several times with DMF and ethanol, respectively.

#### 3.2.2. Synthesis of Mn-DMF-0.05

C_10_H_14_MnO_4_ (0.03 mmol) and C_6_H_7_O_3_P (0.05 mmol) were dissolved in 5 mL DMF and stirred at room temperature. The mixture was dispensed into a Teflon-lined stainless-steel autoclave. The autoclave was maintained at 120 °C for 12 h, and then naturally cooled to room temperature. The resulting precipitate was thoroughly washed several times with DMF and ethanol, respectively.

#### 3.2.3. Synthesis of Mn-DMF-0.2

C_10_H_14_MnO_4_ (0.2 mmol) and C_6_H_7_O_3_P (0.2 mmol) were dissolved in 5 mL DMF and stirred at room temperature. The mixture was dispensed into a Teflon-lined stainless-steel autoclave. The autoclave was maintained at 120 °C for 12 h, and then naturally cooled to room temperature. The resulting precipitate was thoroughly washed several times with DMF and ethanol, respectively.

#### 3.2.4. Preparation of Mn-0.05-X

Among the Mn-DMF-0.05 precursors synthesized above, the samples with nano-strip morphologies were selected and calcined in air at 150, 250, 350, and 550 °C. The heating rate was maintained at 1 °C min^−1^, then the reaction stopped and the temperature was allowed to reduce naturally. The obtained products were denoted as M-0.05-x, where x represents the activation temperatures.

### 3.3. Material Characterization

The morphological features were characterized by scanning electron microscopy (Zeiss, Oberkochen, Germany), high resolution transmission electron microscopy (FEI part of Thermo Fisher Scientific now), Hillsboro, United States), and energy dispersive spectroscopy (EDS) mapping. X-ray diffraction (XRD) patterns were examined on a Bruker D8 Advanced X-ray diffractometer (Cu K_α_ radiation: λ = 0.15406 nm). The chemical states were analyzed using X-ray photoelectron spectroscopy (XPS) with monochromatic Al K_α_ excitation under vacuum higher than 1 × 10^−7^ Pa. In addition, a Fourier Transform-Infrared Radiation (FT-IR) measurement was performed on a BRUKER-EQUINOX-55 IR spectrophotometer. The thermogravimetric analysis (TGA) was performed under air atmosphere with a heating rate of 10 °C min^−1^ by using a Pyris 1 TGA thermogravimetric analyzer. All electrochemical measurements were carried out using a CHI 660E instrument.

### 3.4. Fabrication of the Electrodes in a Traditional Three-Electrode System

The electrochemical measurements were carried out with a CHI760e working station in 3.0 M KOH solution at room temperature. Galvanostatic charge–discharge (GCD), cyclic voltammetry (CV), and electrochemical impedance spectroscopy (EIS) methods were used to investigate the capacitive properties of the Mn-MOP and Mn-0.05-x electrodes. The EIS measurements were conducted in the frequency range of 100 kHz to 0.01 Hz at the open circuit voltage.

For the three-electrode cell, the working electrode was made by mixing the active materials, acetylene black, and polytetrafluoroethylene at a weight ratio of 80:15:5. The slurry was coated on a piece of nickel foam (~1 cm^2^), which was then pressed into a thin foil at a pressure of 10 MPa. The typical mass loading of the electrode material was 1.0 mg. A platinum electrode and Hg/HgO electrode were used as counter and reference electrodes.

### 3.5. Fabrication of the Aqueous Electrochemical Energy Storage Device

Aqueous electrochemical energy storage devices were assembled by employing the Mn-0.05-550 as a positive electrode, and activated carbon was used as a negative electrode. The mass loading for the negative electrode was determined by balancing the charges stored in each electrode. Generally, the charges stored by positive and negative electrodes can be determined by *q_+_* = *C_+_* × ∆*E_+_* × *m_+_* and *q_–_* = *C_–_* × ∆*E_–_* × *m_–_*, where *C_+_*, *C*_–_ represent the specific capacitance of the positive electrode and the negative electrode (F g^−1^), respectively; ∆*E* is the potential range (V); *m_+_*, *m_–_* is the weight of the active material in the positive electrode and the negative electrode (g), respectively. The charges are balanced by the equation of *q_+_* = *q_–_*, where *q_+_* and *q_–_* represent the charges stored in the positive and negative electrodes, respectively. Therefore, *m_+_*/*m_–_* = *C_–_* × ∆*E_–_*/*C_+_* × ∆*E_+_*. The specific capacitance of the purchased activated carbon electrode was 168 F g^−1^ when the current density was 1 A g^−1^. The electrochemical performance of the devices was measured at room temperature in a two-electrode electrochemical full cell. The electrolyte was 3.0 M KOH aqueous solution.

### 3.6. Fabrication of the Solid-State Flexible Electrochemical Energy Storage Device

The positive and negative electrodes were prepared using the same method as the electrodes in the aqueous device. The PVA/KOH gel electrolyte was prepared as follows: 1.52 g PVA was added to 15 mL deionized water and the as-obtained solution was heated to 75 °C for 30 min, then 5 mL KOH aqueous solution was added dropwise into the gel solution under stirring. The positive and negative electrodes were placed on different sides of the PET substrate, and then coated with the gel solution covering the active materials. After the excess water was vaporized, the positive and negative electrodes, including electrolyte, were sandwiched between two pieces of PET substrate. Then, the flexible solid-state device was fabricated.

### 3.7. Calculation

The mass-specific capacitance (C/F g^−1^) of the device can also be calculated using:

*C* = *Q*/(*m* × ∆*V*) = ∫*I*d*t*/(*m* × ∆*V*) = *I* × *t_discharge_*/(*m* × ∆*V*) (1)

where *m* is the mass of the activated materials, *I* is the discharge current, *t_discharge_* is the discharge time, and ∆*V* is the potential drop during discharge.

The area-specific capacitance (C/mF cm^−2^) of the device can also be calculated using:

*C* = *Q*/(*A* × ∆*V*) = ∫*I*d*t*/(*A* × ∆*V*) = *I* × *t_discharge_*/(*A* × ∆*V*) (2)

where *A* is the surface area of the device, *I* is the discharge current, *t_discharge_* is discharge time, and ∆*V* is the potential drop during discharge.

## 4. Conclusions

In summary, Mn-MOPs with three different morphologies were prepared through the solvothermal method. When molar ratio of reactants was 3:5, the morphology of Mn-MOP is a nano-strip. Meanwhile, the Mn-0.05-550 sample retained the nano-strip morphology of the Mn-DMF-0.05 precursor. As an electrode material of SC, Mn-0.05-550 had a better specific capacitance of 230.9 F g^−1^ at 0.5 A g^−1^, and possessed good cycle stability. Additionally, it was particularly noteworthy that the assembled aqueous/solid-state device had a good rate capability and superb cycling performance. The design of the Mn-0.05-550//AC flexible aqueous/solid-state device showed potential in the fields of portable and flexible electronics.

## Data Availability

The data that support the findings of this study are available from the corresponding author upon reasonable request.

## Supplementary Materials

The following supporting information can be downloaded at: https://www.mdpi.com/article/10.3390/molecules29174186/s1, Figure S1: SEM images of (a) Mn-DMF-0.15; (b) Mn-DMF-0.05; (c) Mn-DMF-0.2; TEM images of (d) Mn-DMF-0.15; (e) Mn-DMF-0.05; (f) Mn-DMF-0.2; Figure S2: (a) XRD patterns of Mn-DMF-x; (b) FT-IR spectra of Mn-DMF-x; Figure S3: TG curves of Mn-DMF-0.05; Figure S4: (a) N_2_ adsorption-desorption isotherms and (d) pore size distribution curves of Mn-0.05-550; Figure S5: The CV curves with a scan rate at 20 mV s^−1^ of active materials in a three-electrode cell in 3.0 M KOH aqueous solution at different potentials: (a) Mn-DMF-0.15; (b) Mn-DMF-0.05; (c) Mn-DMF-0.2; (d) Mn-0.05-150; (e) Mn-0.05-250; (f) Mn-0.05-350; Figure S6: The CV curves of active materials in a three-electrode cell in 3.0 M KOH aqueous solution at different scan rates: (a) Mn-DMF-0.15; (b) Mn-DMF-0.05; (c) Mn-DMF-0.2; (d) Mn-0.05-150; (e) Mn-0.05-250; (f) Mn-0.05-350; Figure S7: CV curve with the capacitive fraction shown by the shaded area of Mn-DMF-0.15 at various scan rates in a three-electrode cell. (a) 5 mV s^−1^; (b) 10 mV s^−1^; (c) 20 mV s^−1^; (d) 50 mV s^−1^; (e) 100 mV s^−1^; (f) the percent of capacitive contribution of the Mn-DMF-0.15; Figure S8: CV curve with the capacitive fraction shown by the shaded area of Mn-DMF-0.05 at various scan rates in a three-electrode cell. (a) 5 mV s^−1^; (b) 10 mV s^−1^; (c) 20 mV s^−1^; (d) 50 mV s^−1^; (e) 100 mV s^−1^; (f) the percent of capacitive contribution of the Mn-DMF-0.05; Figure S9: CV curve with the capacitive fraction shown by the shaded area of Mn-DMF-0.2 at various scan rates in a three-electrode cell. (a) 5 mV s^−1^; (b) 10 mV s^−1^; (c) 20 mV s^−1^; (d) 50 mV s^−1^; (e) 100 mV s^−1^; (f) the percent of capacitive contribution of the Mn-DMF-0.2; Figure S10: CV curve with the capacitive fraction shown by the shaded area of Mn-0.05-150 at various scan rates in a three-electrode cell. (a) 5 mV s^−1^; (b) 10 mV s^−1^; (c) 20 mV s^−1^; (d) 50 mV s^−1^; (e) 100 mV s^−1^; (f) the percent of capacitive contribution of the Mn-0.05-150; Figure S11: CV curve with the capacitive fraction shown by the shaded area of Mn-0.05-250 at various scan rates in a three-electrode cell. (a) 5 mV s^−1^; (b) 10 mV s^−1^; (c) 20 mV s^−1^; (d) 50 mV s^−1^; (e) 100 mV s^−1^; (f) the percent of capacitive contribution of the Mn-0.05-250; Figure S12: CV curve with the capacitive fraction shown by the shaded area of Mn-0.05-350 at various scan rates in a three-electrode cell. (a) 5 mV s^−1^; (b) 10 mV s^−1^; (c) 20 mV s^−1^; (d) 50 mV s^−1^; (e) 100 mV s^−1^; (f) the percent of capacitive contribution of the Mn-0.05-350.; Figure S13: The GCD curves of active materials in a three-electrode cell in 3.0 M KOH aqueous solution at different current densities: (a) Mn-DMF-0.15; (b) Mn-DMF-0.05; (c) Mn-DMF-0.2; (d) Mn-0.05-150; (e) Mn-0.05-250; (f) Mn-0.05-350; Figure S14: The EIS of the active materials in a three-electrode cell in 3.0 M KOH aqueous solution at room temperature; Figure S15: Specific capacitance at different current densities of Mn-0.05-550//AC solid-state flexible device; Table S1: Comparison of supercapacitors performance of manganese-based compounds electrodes. References [37,38,39,40,41,42,43,44] are cited in the supplementary materials.
