# Supplementary material for: Controllable Synthesis of Manganese Organic Phosphate with Different Morphologies and Their Derivatives for Supercapacitors
Source: Molecules. 2024 Sep 4;29(17):4186. doi: 10.3390/molecules29174186 (PMC11397101; doi:10.3390/molecules29174186)
Supplement: Supplementary file 1 [file molecules-29-04186-s001.zip › molecules-3183164-supplementary.pdf]

molecules

Supporting Information

Article

Controllable Synthesis of Manganese Organic Phosphate with Different Morphologies and Their Derivatives for Supercapacitors

Jingwen Zhao †, Qingling Jing †, Ting Zhou, Xinhuan Zhang, Wenting Li * and Huan Pang *

School of Chemistry and Chemical Engineering, Yangzhou University; Yangzhou 225009, China; 13773809781@163.com (J.Z.); jql971122lh@163.com (Q.J.); 18251947900@163.com (T.Z.); 19352670640@163.com (X.Z.)

* Correspondence: wtlichem@yzu.edu.cn (W.L.); huanpangchem@hotmail.com (H.P.)

† These authors contributed equally to this work.

1. Supplementary data section

The morphology of these products was characterized by scanning electron microscopy (SEM) and transmission electron microscopy (TEM). Figure S1a reveals that Mn-DMF-0.15 had a unique morphology of palm leaves, with the bottom of the nanorods connected to each other and the top cracked (see Figure S1d), showing a dot-like radiation distribution. The SEM (Figure S1b) and TEM (Figure S1e) image of Mn-DMF-0.05 show that nanoribbons distributed evenly. As shown in Figure S1c, f, the morphology of Mn-DMF-0.2 was regular rectangular nanosheets with uniform distribution. From these SEM and TEM images, we found that the size of Mn-MOP decreased with the increase of the molar ratio of manganese ions to ligand, which was due to the change in the molar ratio of metal salt and ligand. The X-ray diffraction (XRD) patterns were carried out to study the crystal structures of Mn-MOP, as shown in Figure S2a. The XRD patterns of Mn-MOP show that the samples displayed good crystallinity. Although Mn-DMF-0.15, Mn-DMF-0.05, and Mn-DMF-0.2 had different morphologies, they had the same crystal structure. The Mn-MOP exhibited the main peaks at 6.1° and 12.3° from the (010) and (020) faces, respectively. What's more, we employed the Fourier Transform Infrared Spectroscopy (FT-IR) to confirm the chemical compositions and chemical bonding. It can be seen from the FT-IR spectra, as shown in Figure S2b, three kinds of Mn-MOP with different morphologies had the same chemical composition and chemical bond. In the FT-IR spectra, Mn-MOP displayed a strong peak at near 3470 cm^{-1}, which was the absorption peak of -OH bond. The stretching vibration peaks of the benzene ring skeleton were around 1436 and 1596 cm^{-1}, and the absorption peak located at 3052 cm^{-1} arose from the stretching vibration of the unsaturated C-H bond on the benzene ring. Meanwhile, the absorption peaks at 696 and 721 cm^{-1} in the spectrum indicate that the benzene ring was monosubstituted. In addition, the absorption peak located at approximately 570 cm^{-1} was the vibration absorption peak of the Mn–O bond. Furthermore, the vibration absorption peak of the P–O bond were located at about 981 and 1089 cm^{-1}. The results of XRD and FT-IR indicate that we have successfully synthesized Mn-MOP.

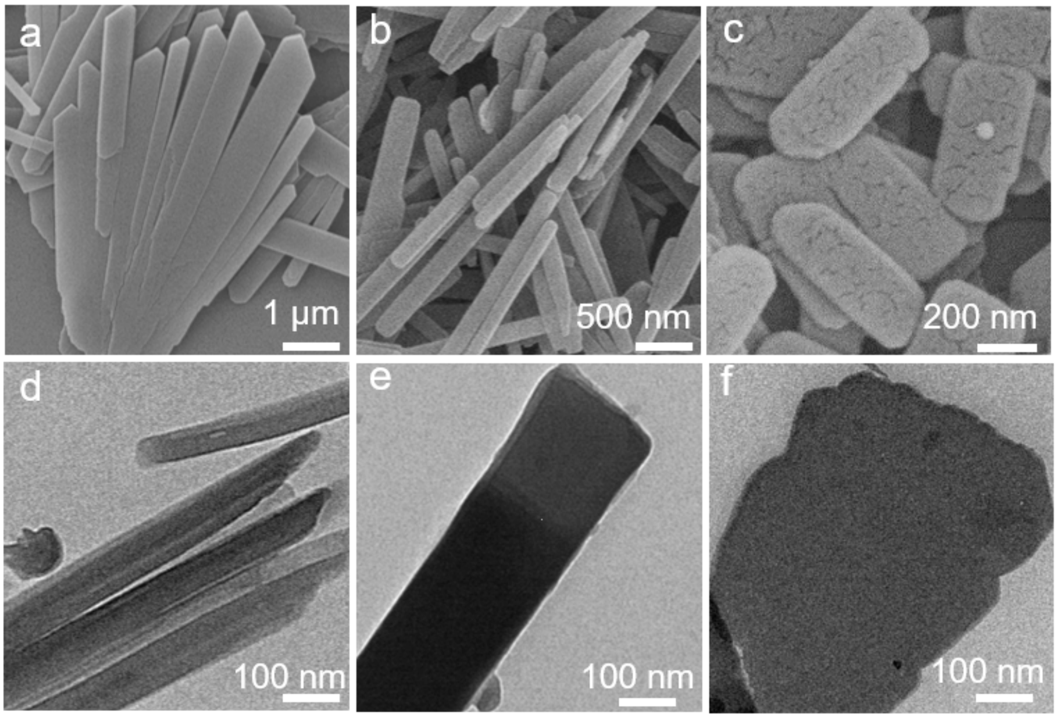

Figure S1. SEM images of **(a)** Mn-DMF-0.15; **(b)** Mn-DMF-0.05; **(c)** Mn-DMF-0.2; TEM images of **(d)** Mn-DMF-0.15; **(e)** Mn-DMF-0.05; **(f)** Mn-DMF-0.2;.

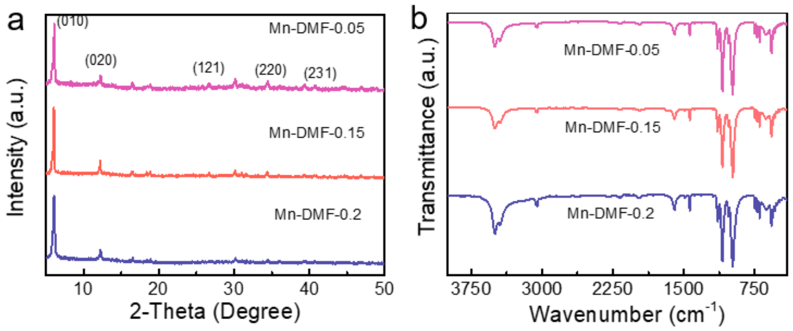

Figure S2. **(a)** XRD patterns of Mn-DMF-x; **(b)** FT-IR spectra of Mn-DMF-x.

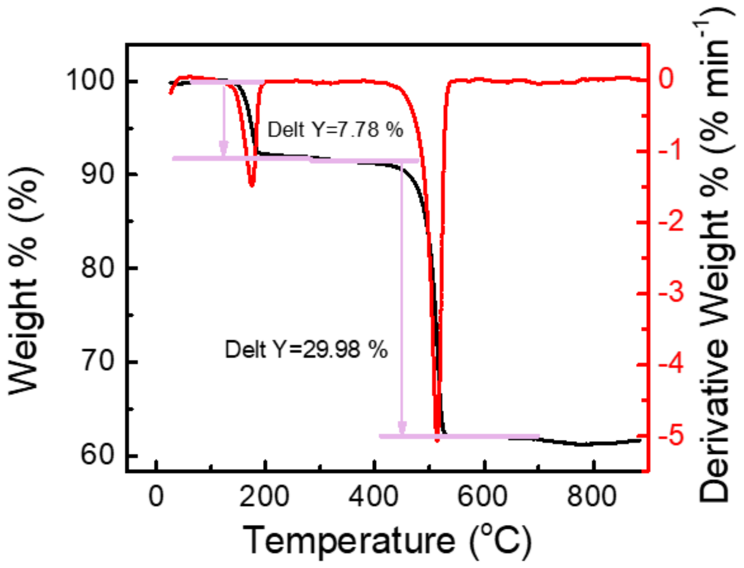

Figure S3. TG curves of Mn-DMF-0.05.

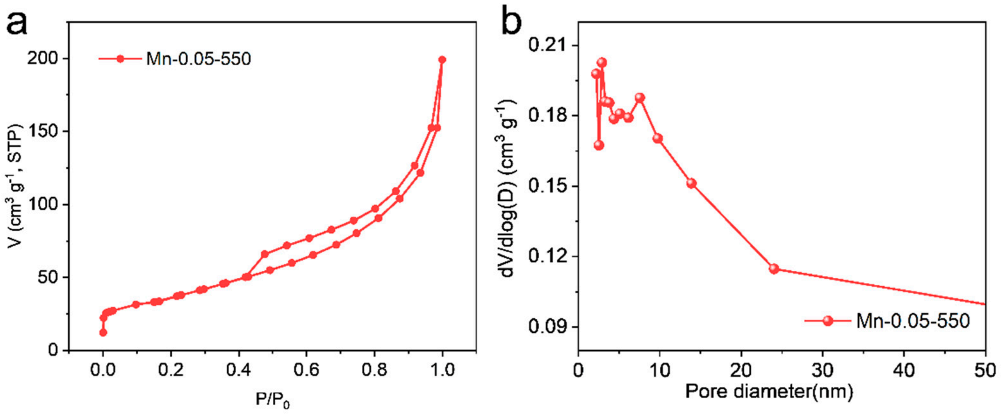

Figure S4. (a) N_2 adsorption-desorption isotherms and **(b)** pore size distribution curves of Mn-0.05-550.

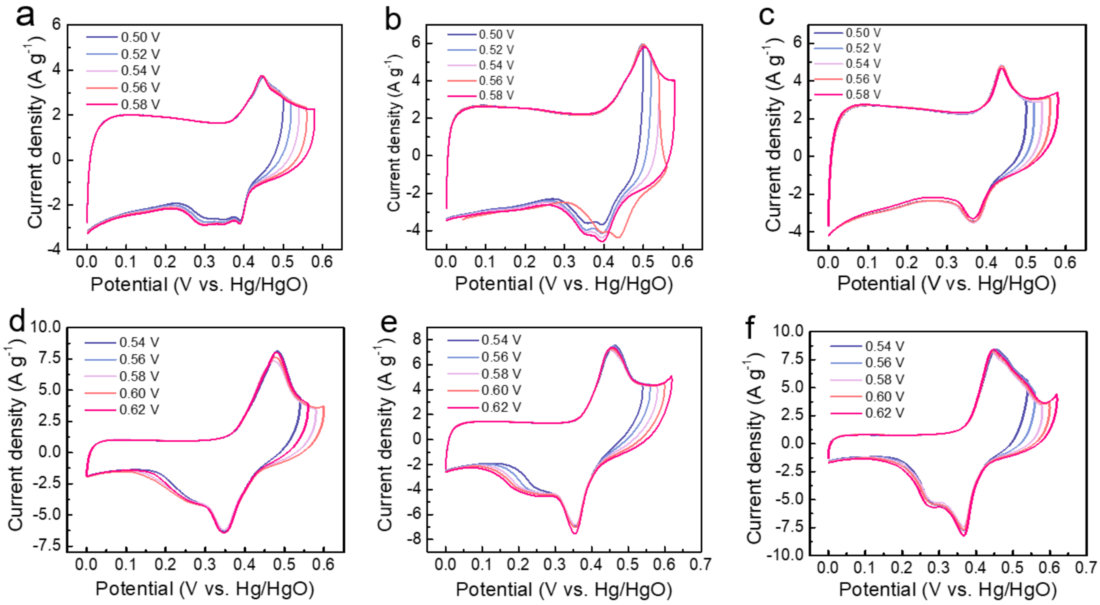

Figure S5. The CV curves with a scan rate at 20 mV s^{-1} of active materials in a three-electrode cell in 3.0 M KOH aqueous solution at different potentials: a) Mn-DMF-0.15; b) Mn-DMF-0.05; c) Mn-DMF-0.2; d) Mn-0.05-150; e) Mn-0.05-250; f) Mn-0.05-350.

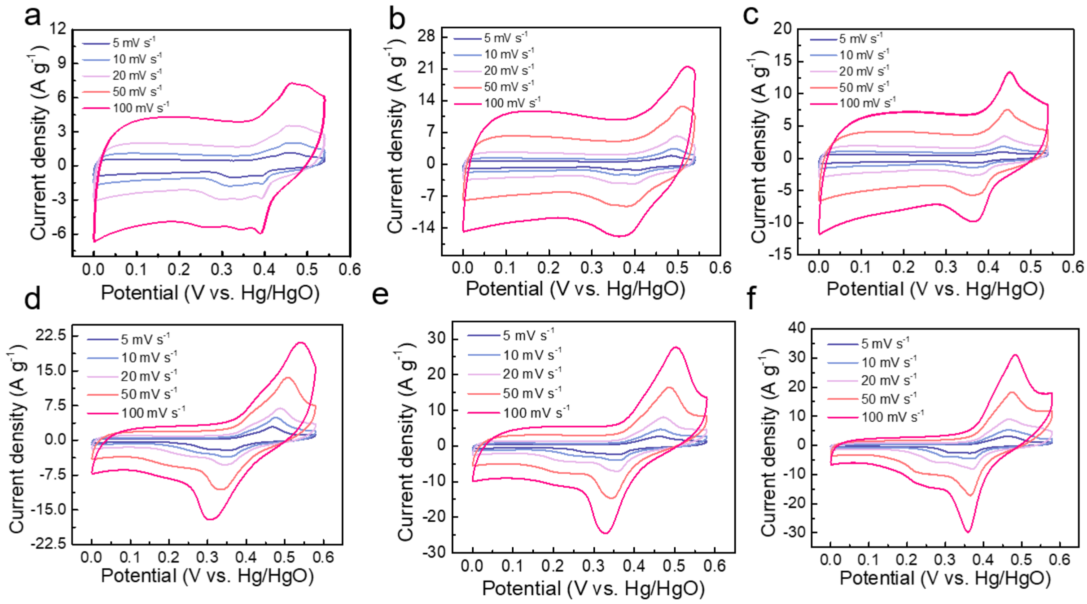

Figure S6. The CV curves of active materials in a three-electrode cell in 3.0 M KOH aqueous solution at different scan rates: a) Mn-DMF-0.15; b) Mn-DMF-0.05; c) Mn-DMF-0.2; d) Mn-0.05-150; e) Mn-0.05-250; f) Mn-0.05-350.

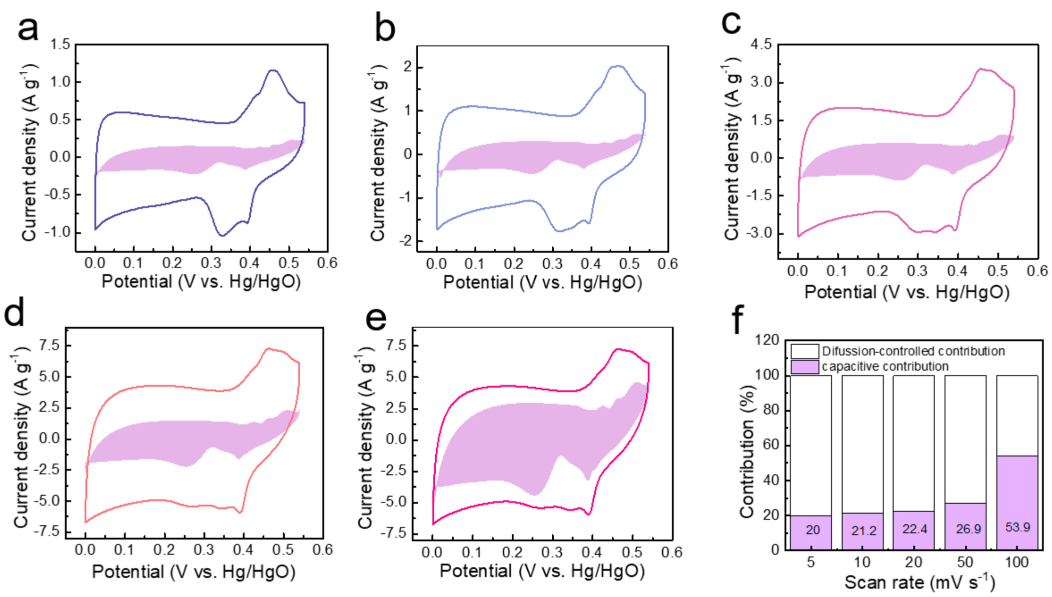

Figure S7. CV curve with the capacitive fraction shown by the shaded area of Mn-DMF-0.15 at various scan rates in a three-electrode cell. a) 5 mV s^{-1}; b) 10 mV s^{-1}; c) 20 mV s^{-1}; d) 50 mV s^{-1}; e) 100 mV s^{-1}; f) the percent of capacitive contribution of the Mn-DMF-0.15.

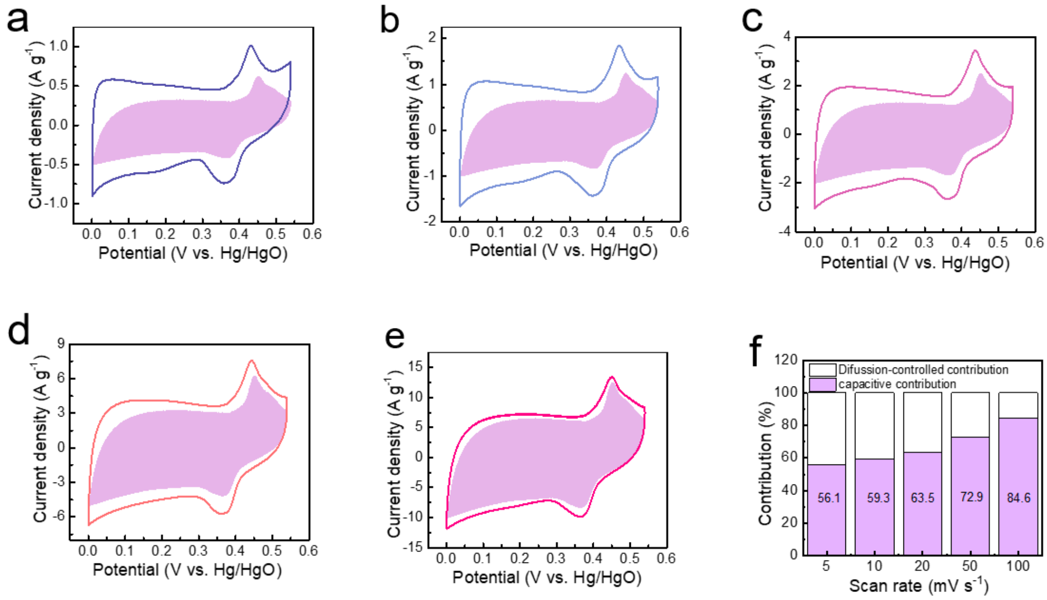

Figure S8. CV curve with the capacitive fraction shown by the shaded area of Mn-DMF-0.05 at various scan rates in a three-electrode cell. a) 5 mV s^{-1}; b) 10 mV s^{-1}; c) 20 mV s^{-1}; d) 50 mV s^{-1}; e) 100 mV s^{-1}; f) the percent of capacitive contribution of the Mn-DMF-0.05.

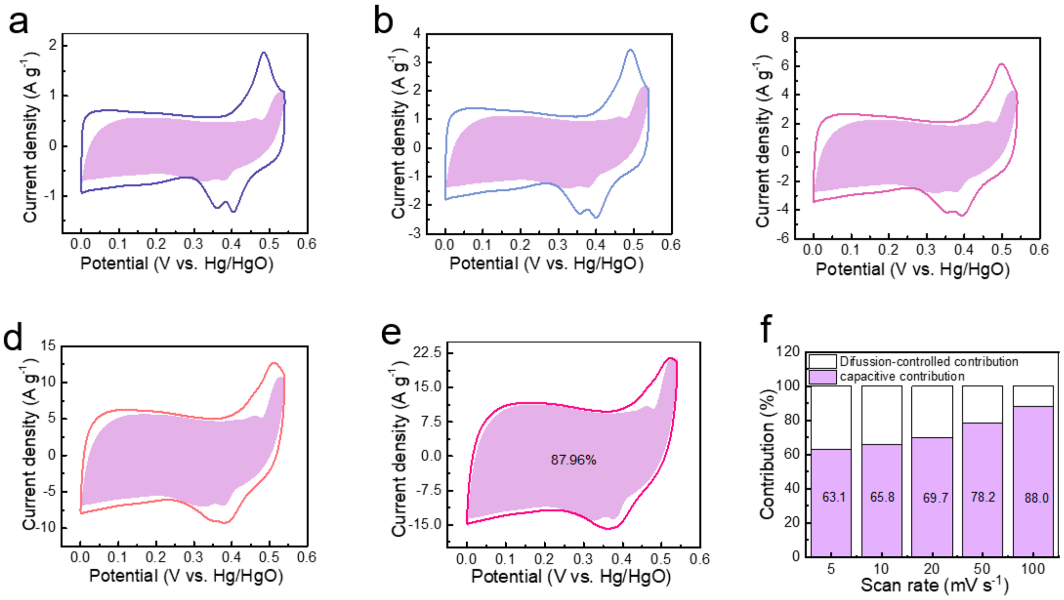

Figure S9. CV curve with the capacitive fraction shown by the shaded area of Mn-DMF-0.2 at various scan rates in a three-electrode cell. a) 5 mV s^{-1}; b) 10 mV s^{-1}; c) 20 mV s^{-1}; d) 50 mV s^{-1}; e) 100 mV s^{-1}; f) the percent of capacitive contribution of the Mn-DMF-0.2.

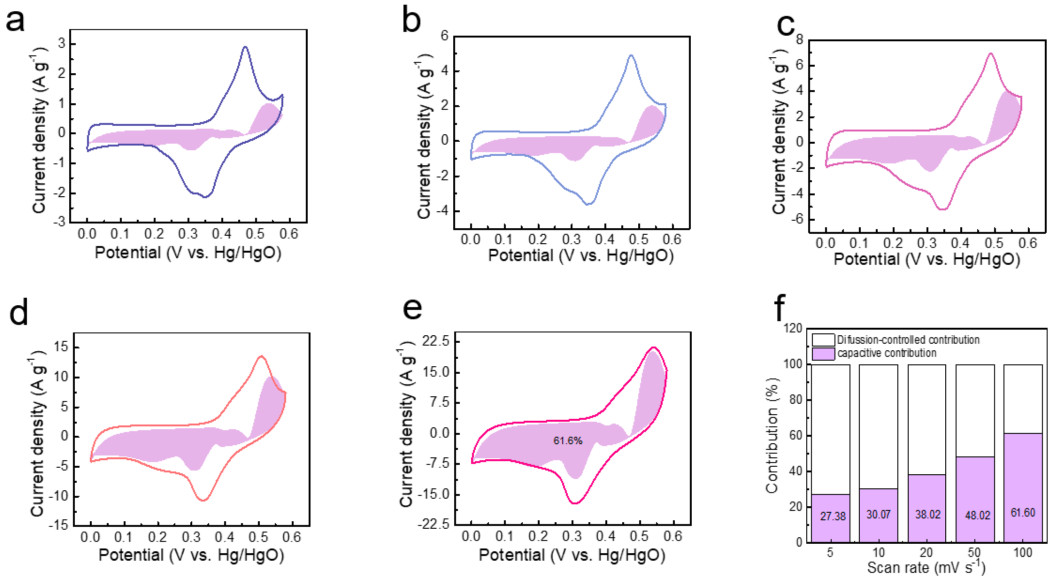

Figure S10. CV curve with the capacitive fraction shown by the shaded area of Mn-0.05-150 at various scan rates in a three-electrode cell. a) 5 mV s^{-1}; b) 10 mV s^{-1}; c) 20 mV s^{-1}; d) 50 mV s^{-1}; e) 100 mV s^{-1}; f) the percent of capacitive contribution of the Mn-0.05-150.

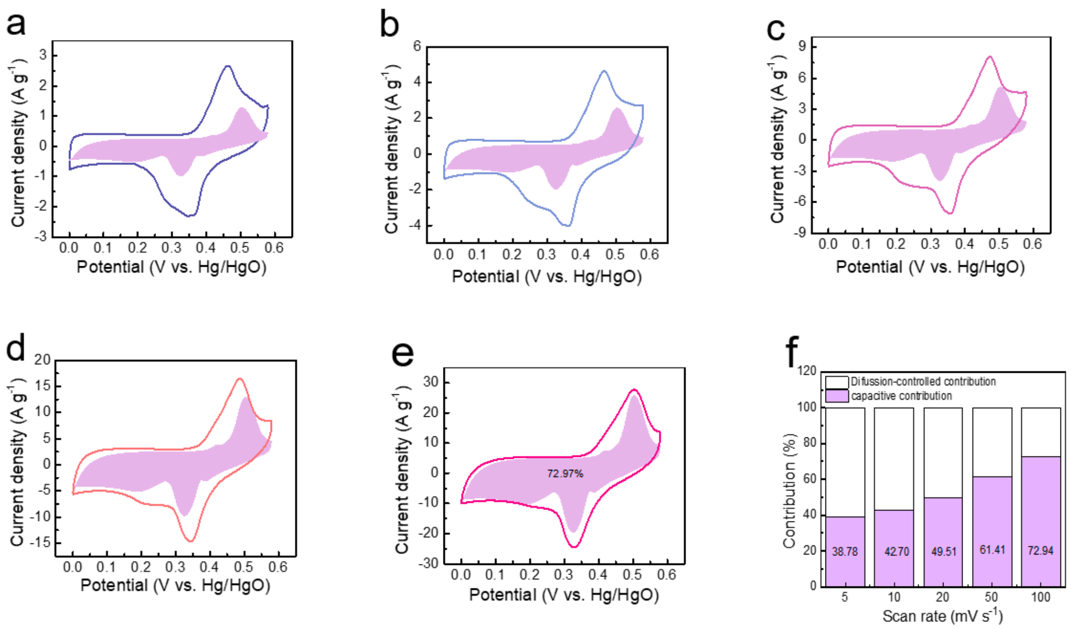

Figure S11. CV curve with the capacitive fraction shown by the shaded area of Mn-0.05-250 at various scan rates in a three-electrode cell. a) 5 mV s^{-1}; b) 10 mV s^{-1}; c) 20 mV s^{-1}; d) 50 mV s^{-1}; e) 100 mV s^{-1}; f) the percent of capacitive contribution of the Mn-0.05-250.

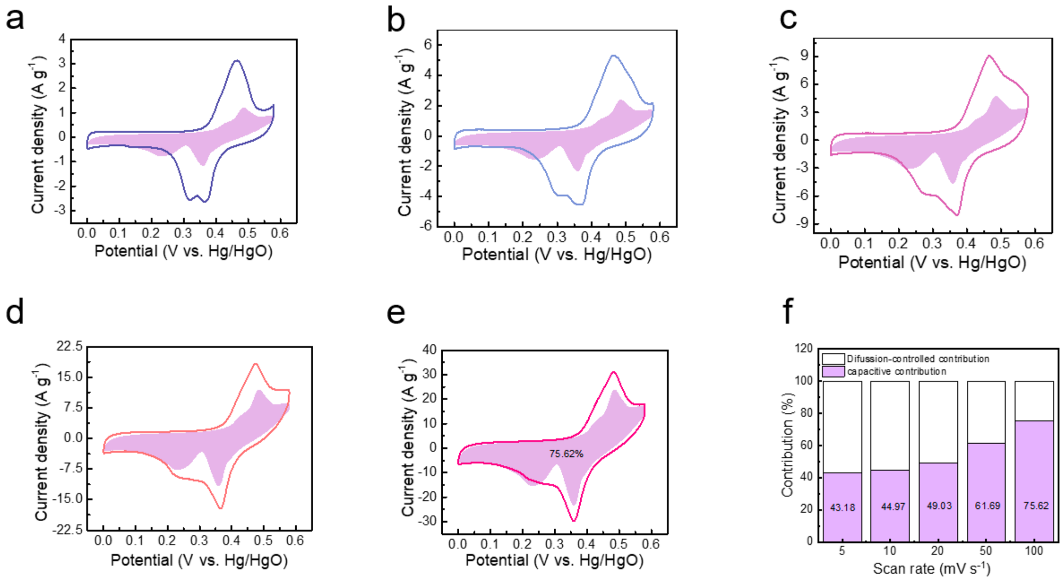

Figure S12. CV curve with the capacitive fraction shown by the shaded area of Mn-0.05-350 at various scan rates in a three-electrode cell. a) 5 mV s^{-1}; b) 10 mV s^{-1}; c) 20 mV s^{-1}; d) 50 mV s^{-1}; e) 100 mV s^{-1}; f) the percent of capacitive contribution of the Mn-0.05-350.

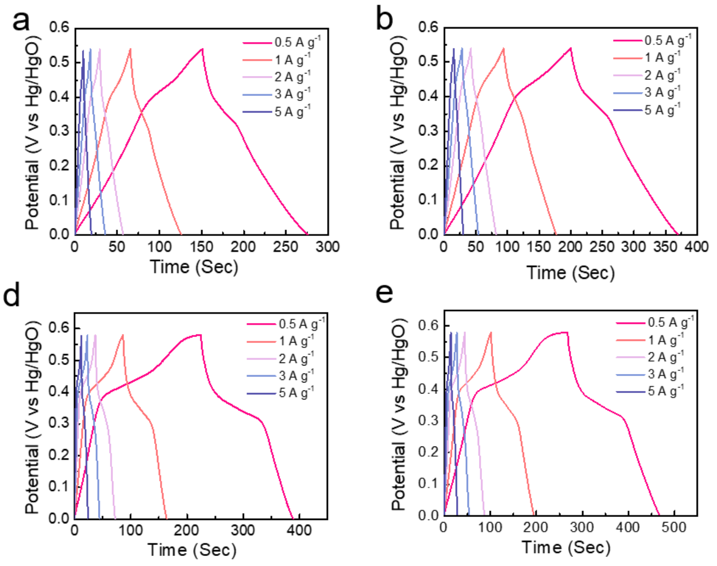

Figure S13. The GCD curves of active materials in a three-electrode cell in 3.0 M KOH aqueous solution at different current densities: a) Mn-DMF-0.15; b) Mn-DMF-0.05; c) Mn-DMF-0.2; d) Mn-0.05-150; e) Mn-0.05-250; f) Mn-0.05-350.

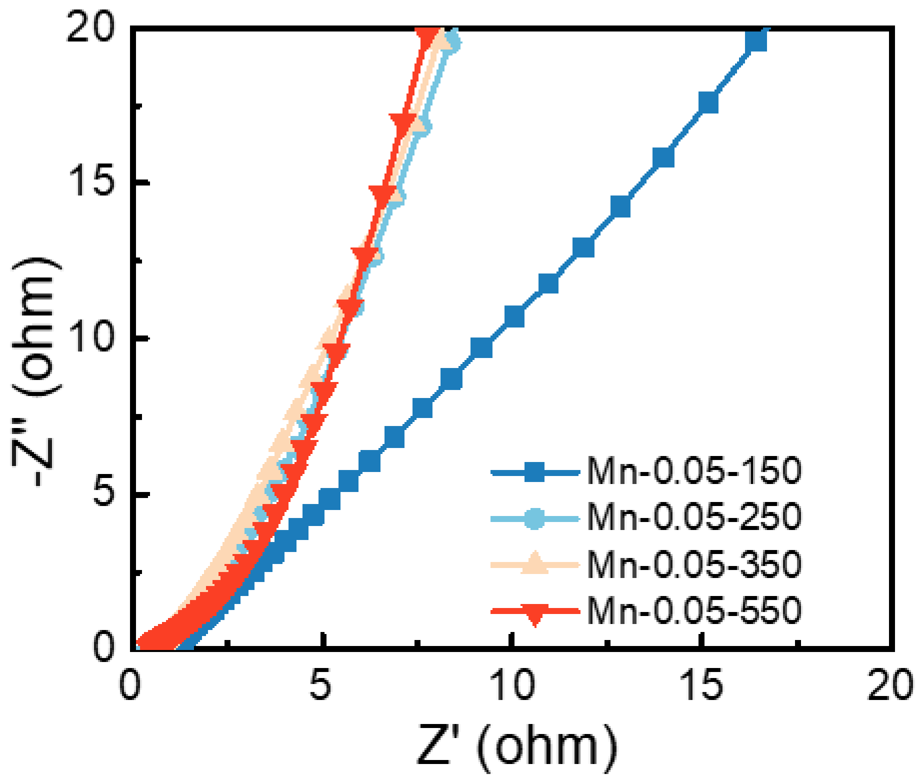

Figure S14. The EIS of the active materials in a three-electrode cell in 3.0 M KOH aqueous solution at room temperature.

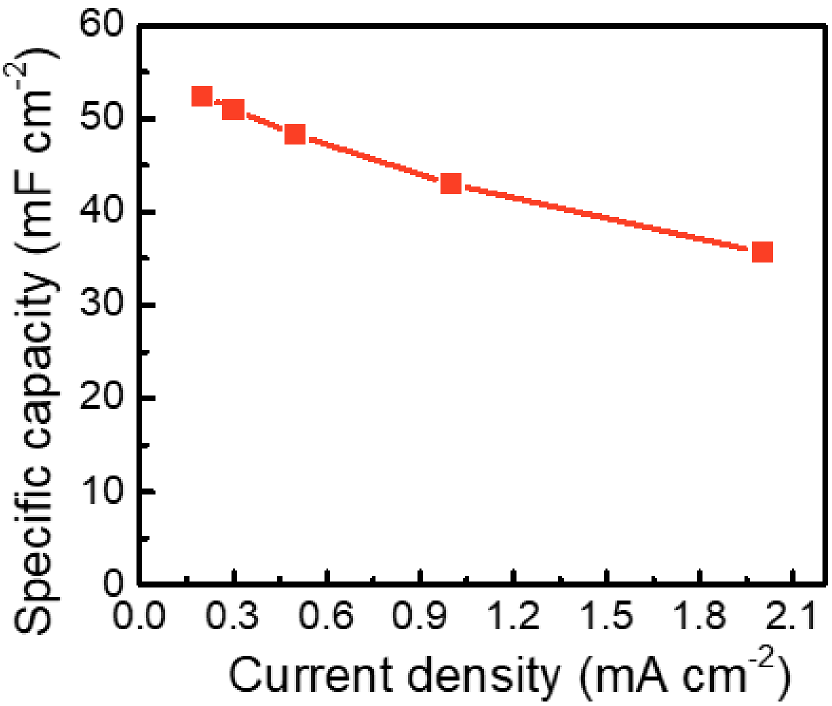

Figure S15. Specific capacitance at different current densities of Mn-0.05-550//AC solid-state flexible device.

Table S1. Comparison of supercapacitors performance of manganese-based compounds electrodes.

Material	Structure	Capacitance	Stability	Cycles	Reference
MP@PPy	Nanosheets	181.2 F g^{-1} at $0.5Ag^{-1}$	93.01%	10000	[1]
$Mn_3(PO_4)_2$	Microrods	145 F g^{-1} at 0.2 mA cm^{-2}	99.50%	7000	[2]
$KMnPO_4 \cdot H_2O$	Submicron particles	329 F g^{-1} at 0.6 mA cm^{-2}	82%	1000	[3]
$MnPO_4 \cdot H_2O$/GO	Nanowires	288 F g^{-1} at 0.625 Ag^{-1}	---	---	[4]
$Mn_3(PO_4)_2 \cdot 3H_2O$/GN	Nanosheets	152 F g^{-1} at $0.5Ag^{-1}$	---	---	[5]
$Mn_3(PO_4)_2$	Nanosheets	203 F g^{-1} at $0.5Ag^{-1}$	91.10%	10000	[6]
$NH_4MnPO_4 \cdot H_2O$	Micro-nanostructures	35 F g^{-1} at 10 mVs^{-1}	---	---	[7]
PANI/MnO_2	Core-shell nanostructure	215 F g^{-1} at $0.3Ag^{-1}$	61%	500	[8]
MnO_2/MnS	Nanorods	305 F g^{-1} at $1Ag^{-1}$	---	---	[9]
α-MnO_2	Nanorods	163.5 F g^{-1} at $0.5Ag^{-1}$	---	---	[10]
Mn-0.05-550	Nano-strip	230.9 F g^{-1} at 0.5 A g^{-1}	84%	3000	This work

References

1. Du, J.L.; Bao, X.; Zhang, W. Bin; Zhang, L.; Guo, Y.W.; Zhou, X.; Zhang, X.L.; Chai, S.S.; Guo, S.B.; Han, X.W.; et al. Electrochemical kinetics of layered manganese phosphate via interfacial polypyrrole chemical binding. *Chem. Electro. Chem* **2022**, *9*, e202101574. https://doi.org/10.1002/celc.202101574.
2. Katkar, P.K.; Marje, S.J.; Pujari, S.S.; Khalate, S.A.; Deshmukh, P.R.; Patil, U.M. Single-Pot Hydrothermal synthesis of manganese phosphate microrods as a cathode material for highly stable flexible solid-state symmetric supercapacitors. *Synth. Met.* **2020**, *267*, 116446. https://doi.org/10.1016/j.synthmet.2020.116446.
3. Priyadharsini, N.; Shanmugavani, A.; Vasylechko, L.; Kalai Selvan, R. Sol-Gel synthesis, structural refinement, and electrochemical properties of potassium manganese phosphate for supercapacitors. *Ion. (Kiel).* **2018**, *24*, 2073–2082. https://doi.org/10.1007/s11581-018-2449-y.
4. Yan, B.; Bin, D.; Ren, F.; Xiong, Z.; Zhang, K.; Wang, C.; Du, Y. Facile synthesis of $mnpo_4{\cdot}h_2o$ nanowire/graphene oxide composite material and its application as electrode material for high performance supercapacitors. *Catal.* **2016**, *6*, 198. https://doi.org/10.3390/catal6120198.
5. Yang, C.; Dong, L.; Chen, Z.; Lu, H. High-performance all-solid-state supercapacitor based on the assembly of graphene and manganese(II) phosphate nanosheets. *J. Phys. Chem. C* **2014**, *118*, 18884–18891. https://doi.org/10.1021/jp504741u.
6. Li, M.; Zhao, M.; Wu, B.; Mourdikoudis, S.; Wei, S.; Oliveira, F.M.; He, J.; Děkanovský, L.; Luxa, J.; Yang, S.; et al. Rational design of crystalline/amorphous nickel manganese phosphate octahydrate heterostructure for high-performance aqueous and all-solid-state asymmetric supercapacitors. *Chem. Eng. J.* **2024**, *482*, 148895. https://doi.org/10.1016/j.cej.2024.148895.
7. Pang, H.; Yan, Z.; Wang, W.; Wei, Y.; Li, X.; Li, J. Template-free controlled fabrication of $nh_4mnpo_4{\cdot}h_2o$ and $mn_2p_2o_7$ micro-nanostructures and study of their electrochemical properties. *Int. J. Electrochem. Sci.* **2012**, *7*, 12340–12353. https://doi.org/10.1016/s1452-3981(23)16549-7.
8. Zhuang, Z.; Wang, W.; Wei, Y.; Li, T.; Ma, M.; Ma, Y. Preparation of polyaniline nanorods/manganese dioxide nanoflowers core/shell nanostructure and investigation of electrochemical performances. *Adv. Compos. Hybrid Mater.* **2021**, *4*, 938–945. https://doi.org/10.1007/s42114-021-00225-0.
9. Rahaman, M.; Islam, M.R.; Islam, M.R. Improved electrochemical performance of defect-induced supercapacitor electrodes based on mns-incorporated mno_2 nanorods. *Nanoscale Adv.* **2024**, *6*, 4103–4110. https://doi.org/10.1039/d4na00085d.
10. Pandit, B.; Goda, E.S.; Abu Elella, M.H.; ur Rehman, A.; Eun Hong, S.; Rondiya, S.R.; Barkataki, P.; Shaikh, S.F.; Al-Enizi, A.M.; El-Bahy, S.M.; et al. One-pot hydrothermal preparation of hierarchical manganese oxide nanorods for high-performance symmetric supercapacitors. *J. Energy Chem.* **2022**, *65*, 116–126. https://doi.org/10.1016/j.jechem.2021.05.028.

Disclaimer/Publisher's Note: The statements, opinions and data contained in all publications are solely those of the individual author(s) and contributor(s) and not of MDPI and/or the editor(s). MDPI and/or the editor(s) disclaim responsibility for any injury to people or property resulting from any ideas, methods, instructions or products referred to in the content.
